# Identification and validation of a novel pyroptosis-related lncRNAs signature associated with prognosis and immune regulation of hepatocellular carcinoma

**DOI:** 10.1038/s41598-022-13046-y

**Published:** 2022-05-25

**Authors:** Zeyu Zhang, Fada Xia, Zhijie Xu, Jinwu Peng, Fanhua Kang, Jianbo Li, Wenqin Zhang, Qianhui Hong

**Affiliations:** 1 Department of Thyroid Surgery, Xiangya Hospital, Central South University, Changsha, 410008 Hunan China; 2 Department of Pathology, Xiangya Hospital, Central South University, Changsha, 410008 Hunan China; 3 National Clinical Research Center for Geriatric Disorders, Xiangya Hospital, Central South University, Changsha, 410008 Hunan China; 4 Department of Pathology, Xiangya Changde Hospital, Changde, 415000 Hunan China

**Keywords:** Cancer genetics, Cancer microenvironment, Tumour biomarkers

## Abstract

Pyroptosis is an inflammatory form of cell death triggered by certain inflammasomes. However, research concerning pyroptosis-related lncRNAs in hepatocellular carcinoma (HCC) remains scarce. This study aims to explore the prognostic pyroptosis-related long non-coding RNAs (lncRNAs) of HCC patients. Data of 373 HCC patients were obtained from the TCGA database. The entire cohort was randomly divided into a training cohort and a validation cohort in a 2:1 ratio. Pyroptosis-related lncRNAs were identified by the Pearson correlation analysis with reported pyroptosis-related genes. LASSO Cox regression was used to construct the signature. A prognostic signature consisting of nine pyroptosis-related lncRNAs was identified, and patients with lower risk scores had a better prognosis than those with higher risk scores. Multivariate Cox regression analysis showed that the signature was an independent risk factor for prognosis in both the training and validation cohorts. In the training cohort, the area under the signature curve reached 0.8043 at 1-year, 0.7878 at 2-year, and 0.8118 at 3-year; in the validation cohort, it reached 0.7315 at 1-year, 0.7372 at 2-year, and 0.7222 at 3-year. Gene set enrichment analysis (GSEA) suggested associations between the signature and several immune-related pathways. The expression of multiple immune checkpoints was also increased in the high-risk group, including PD-1, PD-L1, CTLA4, B7-H3, VSIR, LAG3, and TIGIT. A novel pyroptosis-related lncRNA signature, which may be associated with tumor immunity and potentially serve as an indicator for immunotherapy, has been identified to precisely predict the prognosis of HCC patients.

## Introduction

Hepatocellular carcinoma (HCC), one of the leading causes of cancer-related death, is the most common liver cancer, followed by intrahepatic cholangiocarcinoma^1,2^. Although early-stage HCC can be cured by surgical intervention, many challenges remain in treating the advanced HCC, leading to a poor prognosis, high economic costs, and heavy disease burden^3^. Thus, exploring reliable prognostic factors is vital to preferable individualized management and treatment.

First mentioned in 1992, pyroptosis is an inflammatory form of cell death triggered by certain inflammasomes^4^. Extensive studies have focused on the association between pyroptosis and human diseases, revealing that pyroptosis is related to not only inflammatory diseases but also various cancers, including HCC^5,6^. Additionally, pyroptosis-related genes were previously investigated as well. Lozano-Ruiz et al. described that the absent in melanoma 2 (AIM2) could trigger pyroptosis by activating the inflammasome cascade in HCC^7^.

With advances in sequencing technology, long non-coding RNAs (lncRNAs), a class of RNAs with more than 200 nucleotides, have been found to be functional in most biological and pathological processes^8^. Emerging evidence has suggested the crucial role of lncRNAs in the tumorigenesis and progression of HCC^9^. However, the role of pyroptosis-related lncRNAs in HCC pathogenesis and immune regulation remains underappreciated. Thus, this study was performed to recognize the prognostic pyroptosis-related lncRNAs in HCC, thus providing a better understanding of the prognosis prediction and selection of immunotherapy patients.

## Materials and methods

### Data acquisition

Transcriptome and clinical data of HCC patients, including 373 tumor tissues and 50 normal tissues, were retrieved from the LIHC project of TCGA database (http://cancergenome.nih.gov/). Patients without adequate clinical data were excluded from the analysis. Expression data were normalized to the values of transcripts per kilobase million (TPM) for further analysis.

### Identification of pyroptosis-related lncRNAs

Thirty-three pyroptosis-related genes were obtained from Ye’s reports (Supplementary Material 1)^10^. Pearson correlation test was used to calculate the correlations between lncRNAs and pyroptosis-related genes. Based on the cut-off criteria of Pearson correlation coefficient > 0.3, these lncRNAs were considered as candidate pyroptosis-related lncRNAs.

### Construction and validation of the prognostic pyroptosis-related lncRNAs signature

The cohort was randomly divided into a training cohort and a validation cohort in a 2:1 ratio. The data from the training cohort were used to construct the prognostic pyroptosis-related lncRNAs signature, while the other cohort was used for validation. Univariate Cox regression analysis was used to identify the prognostic pyroptosis-related lncRNAs. Subsequently, the least absolute shrinkage and selection operator (LASSO) Cox regression was used to construct the signature by R packages (glmnet and survival) as follows: risk score = expression of lncRNA1 × β1lncRNA1 + expression of lncRNA2 × β2lncRNA2 + …expression of lncRNAn × βnlncRNAn. The two cohorts were further divided into the low-risk and high-risk groups, respectively. Survival analysis and time-dependent ROC curves were performed to investigate the prognostic value. Moreover, multivariate Cox regression of available patient characteristics was performed to reconfirm the prognostic value of the signature. Additionally, a nomogram was constructed to predict the patient prognosis more precisely.

### The mRNA-lncRNA co-expression network

In order to better demonstrate the associations between pyroptosis-related genes and pyroptosis-related lncRNAs, a co-expression network was constructed. A Sankey diagram was used to illustrate the mRNA-lncRNA relationships.

### Gene set enrichment analysis (GSEA) and subsequent functional enrichment analysis

The tumor tissues were divided into the low-risk group and high-risk group based on risk scores. Differentially expressed genes between groups were identified by the “DEseq2” package with cut-off criteria of false discovery rate < 0.05 and |log_2_foldchange| > 1. After that, these differentially expressed genes were uploaded for GSEA analysis (http://www.broadinstitute.org/gsea)^11^.

### Immunological analysis

The abundance of tumor-infiltrating immune cells in HCC tissues was investigated using the Tumor IMmune Estimation Resource (TIMER) database (https://cistrome.shinyapps.io/timer/)^12^.

### Statistical analysis

R 3.3.0 and Statistical Package for Social Sciences 23.0 (SPSS Inc., Chicago, IL, United States) were used for statistical analysis. One-way analysis of variance (ANOVA) with homogeneous variances was used to analyze differences in immune cell components between the normal and HCC tissues, while Welch’s ANOVA was applied when variances were heterogeneous. Kaplan–Meier curve was used for survival analysis, where a Log-rank test was adopted for comparison between groups.

### Ethics approval and consent to participate

Extra informed consent is not essential for the data were all obtained from public database. The authors cannot assess to any identifying characteristics, which do not distort scientific meaning.

### Research involving human participants and/or animals

This article does not contain any studies with human participants or animals performed by any of the authors.

## Results

### Cohort studies of the training cohort and validation cohort

**Figure 1:**
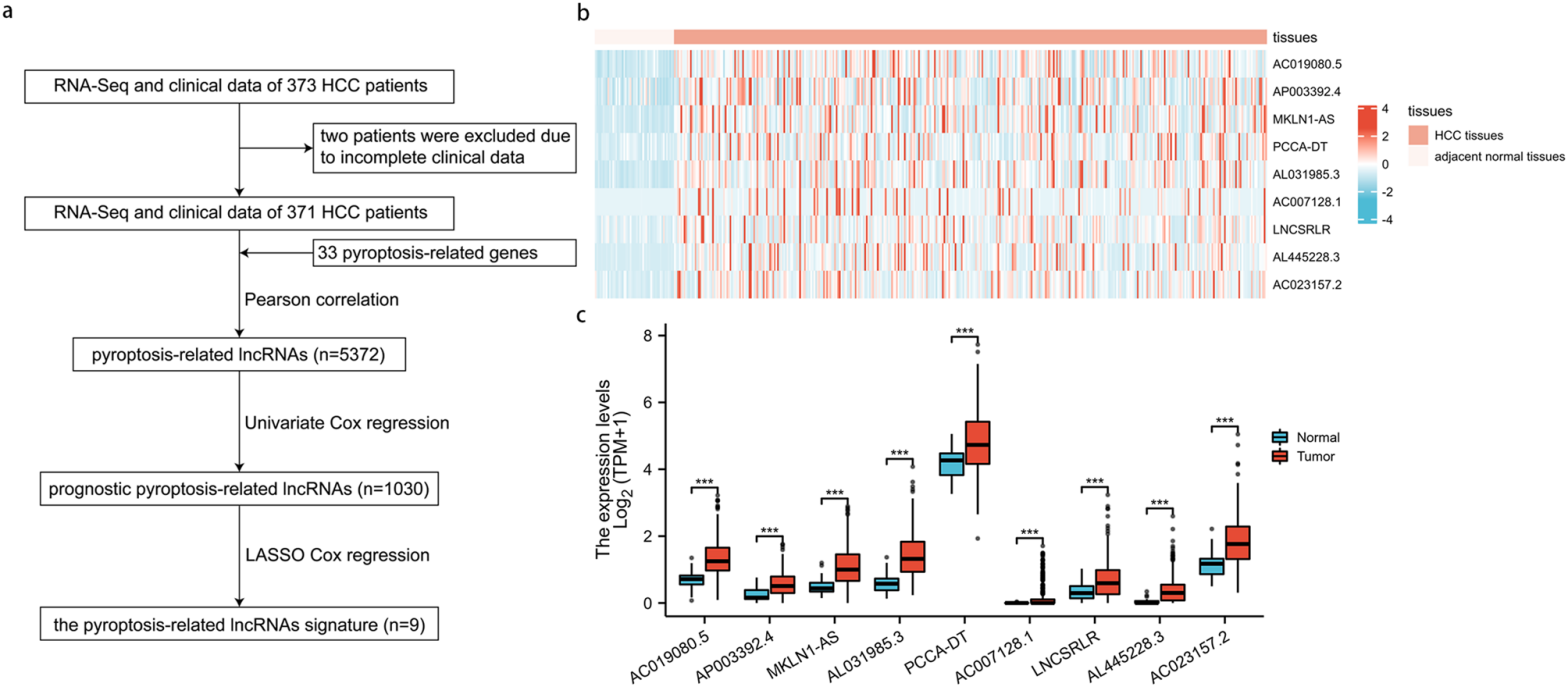
Identification of the pyroptosis-associated lncRNAs in HCC patients. (**a**) The flow chart of constructing the pyroptosis-related lncRNAs signature. (**b**) The heatmap of 9 prognostic pyroptosis-related lncRNAs in HCC tissues and adjacent normal tissues. (**c**) The barplots comparing the 9 prognostic pyroptosis-related lncRNAs between HCC tissues and adjacent normal tissues. HCC, hepatocellular carcinoma. *P < 0.05; **P < 0.01; ***P < 0.001.

A total of 373 HCC patients were enrolled. However, two patients were excluded from this study due to incomplete clinical data (Fig. 1a). Thus, 371 patients were finally included in this study and randomly divided into the training cohort and the validation cohort in a 2:1 ratio. The characteristics of HCC patients in the two cohorts are shown in Table 1, with no statistically significant differences in all characteristics.

**Table 1:** The characteristics of HCC patients in training cohort and validation cohort.

Characteristic	Training cohort (n = 247)	Validation cohort (n = 124)	P value
Age, median (IQR)	61 (51, 69)	61 (52, 68)	0.762
BMI, meidan (IQR)	24.45 (22.15, 28.99)	24.3 (21.35, 28.12)	0.375
**Gender, n (%)**			0.473
Female	77 (31.2%)	44 (35.5%)	
Male	170 (68.8%)	80 (64.5%)	
**Family cancer history, n (%)**			0.639
Yes	75 (33.9%)	37 (37.4%)	
No	146 (66.1%)	62 (62.6%)	
**Race, n (%)**			0.378
American indian or alaska native	1 (0.4%)	1 (0.8%)	
Asian	112 (46.5%)	46 (38.4%)	
Black or african american	10 (4.1%)	7 (5.8%)	
White	118 (49.0%)	66 (55.0%)	
**Alcohol consumption, n (%)**			0.170
Yes	84 (35.9%)	33 (28.0%)	
No	150 (64.1%)	85 (72.0%)	
**Hepatitis B, n (%)**			0.405
Yes	73 (31.2%)	31 (26.3%)	
No	161 (68.8%)	87 (73.7%)	
**Hepatitis C, n (%)**			0.144
Yes	32 (13.7%)	24 (20.3%)	
No	202 (86.3%)	94 (79.7%)	
**T stage, n (%)**			0.767
T1	122 (49.6%)	59 (48.0%)	
T2	59 (24.0%)	35 (28.5%)	
T3	54 (22.0%)	26 (21.1%)	
T4	10 (4.1%)	3 (2.4%)	
TX	1 (0.3%)	0 (0%)	
**N stage, n (%)**			0.736
N0	167 (67.9%)	85 (68.6%)	
N1	2 (0.8%)	2 (1.6%)	
NX	77 (31.3%)	37 (29.8%)	
**M stage, n (%)**			1.000
M0	177 (71.7%)	89 (71.8%)	
M1	3 (1.2%)	1 (0.8%)	
MX	67 (27.1%)	34 (27.4%)	
**Pathologic stage, n (%)**			0.766
Stage I	118 (50.9%)	53 (46.1%)	
Stage II	55 (23.7%)	31 (27.0%)	
Stage III	55 (23.7%)	30 (26.1%)	
Stage IV	4 (1.7%)	1 (0.8%)	
**Neoplasm histologic grade, n (%)**			0.481
G1	33 (13.6%)	22 (17.9%)	
G2	118 (48.6%)	59 (48.0%)	
G3	82 (33.7%)	40 (32.5%)	
G4	10 (4.1%)	2 (1.6%)	
**Child–pugh classification grade, n (%)**			0.172
A	147 (92.5%)	70 (87.5%)	
B	12 (7.5%)	9 (11.3%)	
C	0 (0%)	1 (1.2%)	
**Microvascular invasion, n (%)**			1.000
Yes	72 (34.4%)	37 (34.9%)	
None	137 (65.6%)	69 (65.1%)	

### Identification of prognostic pyroptosis-related lncRNAs

**Figure 2:**
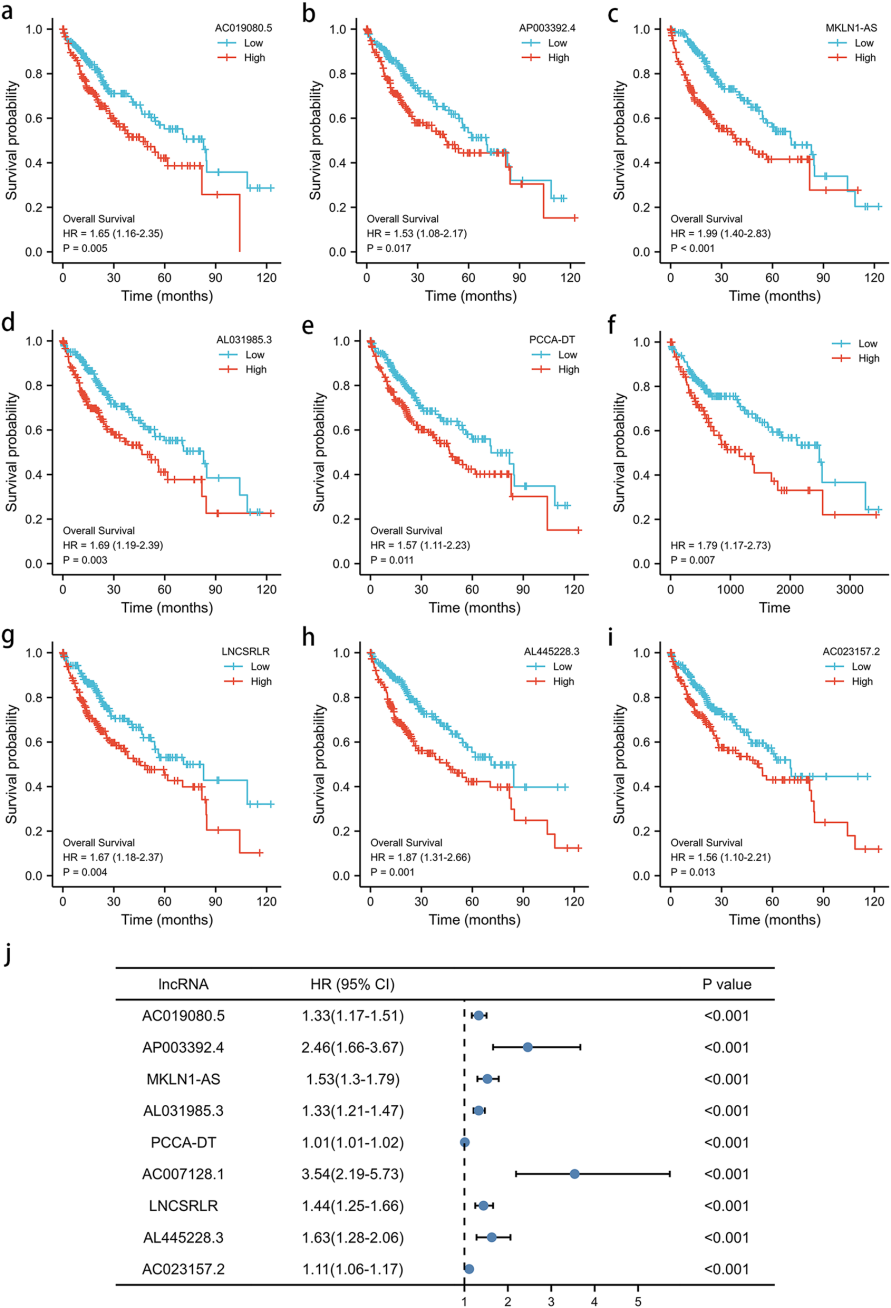
The prognostic value of pyroptosis-associated lncRNAs in HCC patients. (**a**–**i**) Kaplan-Meire curve of AC019080.5 (**a**), AP003392.4 (**b**), MKLN1-AS (**c**), AL031985.3 (**d**), PCCA-DT (**e**), AC007128.1 (**f**), LNCSRLR (**g**), AL445228.3 (**h**), AC023157.2 (**i**) on overall survival of HCC patients. (**j**) Univariate Cox regression of 9 prognostic pyroptosis-related lncRNAs in the training cohort.

Pearson correlation test was performed between lncRNAs and 33 pyroptosis-related genes, and then 5372 lncRNAs were preliminarily identified with PCA analysis in supplement material 2. Subsequently, in the training cohort, the prognostic value was examined by the univariate Cox regression, and 1030 prognostic pyroptosis-related lncRNAs were chosen for further analysis. Using LASSO Cox regression analysis, 9 lncRNAs (AC019080.5, AP003392.4, MKLN1-AS, AL031985.3, PCCA-DT, AC007128.1, LNCSRLR, AL445228.3, AC023157.2) were screened according to lambda.min, and a prognostic signature of the 9 lncRNAs was calculated as follows: risk score = (0.218*AC019080.5 expression) + (0.219*AP003392.4 expression) + (0.103*MKLN1-AS expression) + (0.163*AL031985.3 expression) + (0.003*PCCA-DT expression) + (0.484*AC007128.1 expression) + (0.236*LNCSRLR expression) + (0.161*AL445228.3 expression) + (0.060*AC023157.2 expression). The expression patterns of the 9 lncRNAs in HCC are shown by heatmaps in Fig. 1b and Supplementary Material 3. The 9 lncRNAs were all significantly up-regulated in HCC tissues compared with adjacent normal tissues (Fig. 1c). Their prognostic values are shown in Fig. 2a–i with Kaplan-Miere curves and Fig. 2j with the univariate Cox regression. As shown in Fig. 2a–i, high levels of these candidate lncRNAs are correlated with poor prognosis in HCC patients.

**Figure 3:**
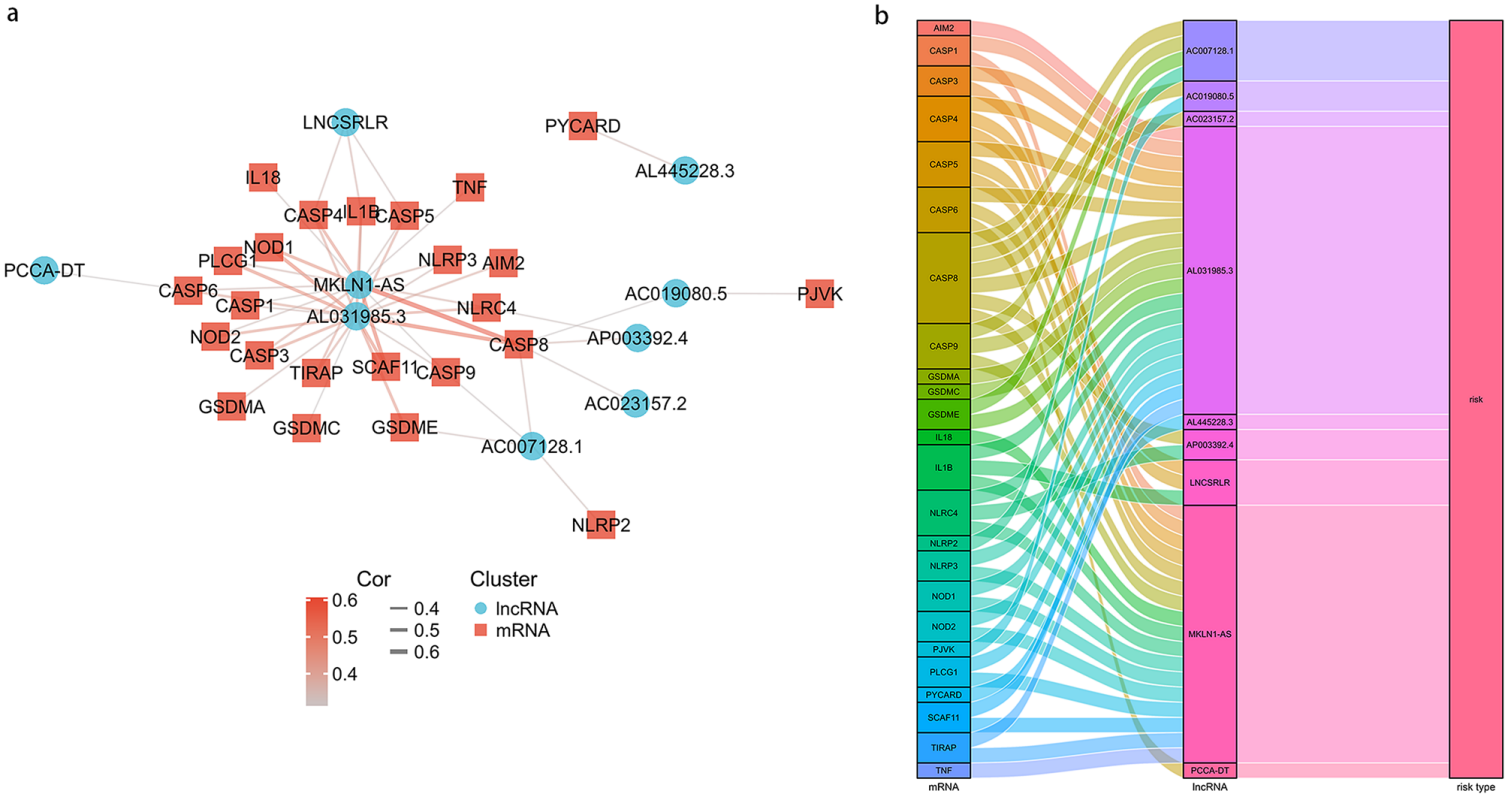
The mRNA-lncRNA co-expression network. (**a**) mRNA-lncRNA co-expression network of the pyroptosis-related genes and the selected pyroptosis-related lncRNAs. (**b**) The Sankey diagram showing the connection degree between the pyroptosis-related lncRNAs and the pyroptosis-related genes.

Then, a co-expression network between the pyroptosis-related genes and pyroptosis-related lncRNAs was constructed to confirm their relationships (Supplementary Material 4). As shown in Fig. 3a and supplement material 5, 24 pyroptosis-related genes and 9 pyroptosis-related lncRNAs are included in the network. It is worth noting that MKLN1-AS and AL031985.3 might be the most likely pyroptosis-related lncRNAs. In addition, the close correlation between pyroptosis-related genes and pyroptosis-related lncRNAs is indicated by the Sankey diagram (Fig. 3b). These findings suggest that the 9 pyroptosis-related lncRNAs might play important roles in HCC.

### Validation of the prognostic pyroptosis-related lncRNA signature

**Figure 4:**
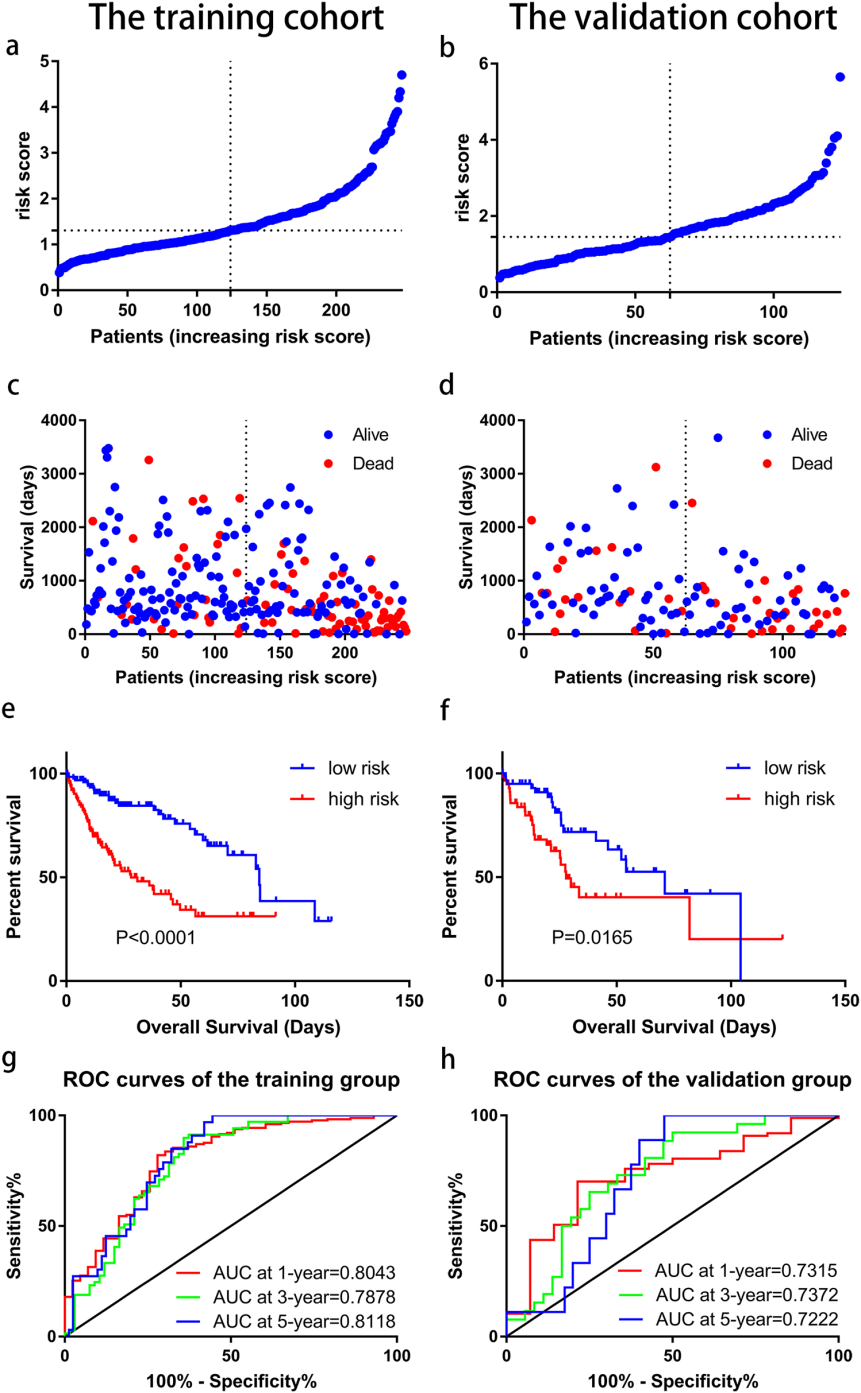
Prognostic analysis of pyroptosis-related lncRNA signature in the training cohort and validation cohort. (**a**) The distribution of the risk scores in the training cohort. (**b**) The distribution of the risk scores in the validation cohort. (**c**) The distributions of overall survival status, overall survival, and risk score in the training cohort. (**d**) The distributions of overall survival status, overall survival, and risk score in the validation cohort. (**e**) Kaplan–Meier curves for the overall survival of patients in the high- and low-risk groups in the training cohort. (**f**) Kaplan–Meier curves for the overall survival of patients in the high- and low-risk groups in the validation cohort. (**g**) AUC of time-dependent ROC curves verified the prognostic accuracy of the risk score in the training cohort. (**h**) AUC of time-dependent ROC curves verified the prognostic accuracy of the risk score in the validation cohort.

The prognostic signature was subsequently validated in the training cohort and validation cohort. The patients in the two cohorts were divided into the low-risk group and high-risk group based on the median risk score. The characteristics of the two groups in the training cohort are compared in Table 2. The high-risk group is proved to be associated with a poorer T stage (p = 0.031), pathologic stage (p = 0.013), and histologic grade (p < 0.001). Figure 4a–d shows that the probability of death is higher in the high-risk group than in the low-risk group in both cohorts. Kaplan–Meier curves of OS show consistent results in both cohorts (Fig. 4e, f), indicating that the survival of HCC-TCGA patients in the high-risk group is significantly worse than that in the low-risk group. A time-dependent receiver operating characteristic (ROC) was performed to investigate values in predicting the patient prognosis (Fig. 4g, h). The area under the curve (AUC) reached 0.8043 at 1-year, 0.7878 at 2-year, and 0.8118 at 3-year in the training cohort, while 0.7315 at 1-year, 0.7372 at 2-year, and 0.7222 at 3-year in the validation cohort. In order to further verify the prognostic value of the signature, multivariate Cox regression analysis was performed in the training cohort (Table 3) and the validation cohort (Table 4). The results show that the risk score could be an independent factor to predict the patient prognosis in the two cohorts.

**Table 2:** Associations between the risk score and characteristics of HCC patients in training cohort.

Characteristic	Low-risk group (n = 124)	High-risk group (n = 123)	P value
Age, median (IQR)	61 (51, 69)	61 (52, 69)	0.924
BMI, meidan (IQR)	25.65 (23.36, 30.12)	23.42 (20.94, 27.45)	< 0.001
**Gender, n (%)**			0.966
Female	38 (30.6%)	39 (31.7%)	
Male	86 (69.4%)	84 (68.3%)	
**Family cancer history, n (%)**			0.736
Yes	38 (35.5%)	37 (32.5%)	
No	69 (64.5%)	77 (67.5%)	
**Race, n (%)**	122	119	0.360
American Indian or Alaska native	0 (0%)	1 (0.8%)	
Asian	56 (45.9%)	56 (47.1%)	
Black or African American	3 (2.5%)	7 (5.9%)	
White	63 (51.6%)	55 (46.2%)	
**Alcohol consumption, n (%)**		118	0.392
Yes	38 (32.8%)	46 (39.0%)	
No	78 (67.2%)	72 (61.0%)	
**Hepatitis B, n (%)**			0.224
Yes	41 (35.3%)	32 (27.1%)	
No	75 (64.7%)	86 (72.9%)	
**Hepatitis C, n (%)**			0.533
Yes	18 (15.5%)	14 (11.9%)	
No	98 (84.5%)	104 (88.1%)	
**T stage, n (%)**			0.031
T1	71 (57.7%)	51 (41.5%)	
T2	29 (23.6%)	30 (24.3%)	
T3	19 (15.4%)	35 (28.5%)	
T4	3 (2.4%)	7 (5.7%)	
TX	1 (0.8%)	0 (0%)	
**N stage, n (%)**			0.945
N0	85 (68.4%)	82 (67.2%)	
N1	1 (0.8%)	1 (0.8%)	
NX	38 (30.6%)	39 (32.0%)	
**M stage, n (%)**			0.711
M0	91 (73.4%)	86 (69.9%)	
M1	1 (0.8%)	2 (1.6%)	
MX	32 (25.8%)	35 (28.5%)	
**Pathologic stage, n (%)**			0.013
Stage I	69 (59.0%)	49 (42.6%)	
Stage II	28 (23.9%)	27 (23.5%)	
Stage III	18 (15.4%)	37 (32.2%)	
Stage IV	2 (1.7%)	2 (1.7%)	
**Neoplasm histologic grade, n (%)**			< 0.001
G1	23 (18.9%)	10 (8.3%)	
G2	65 (53.3%)	53 (43.8%)	
G3	33 (27.0%)	49 (40.5%)	
G4	1 (0.8%)	9 (7.4%)	
**Child–pugh classification grade, n (%)**			0.787
A	86 (93.5%)	61 (91.0%)	
B	6 (6.5%)	6 (9.0%)	
**Microvascular invasion, n (%)**			0.113
Yes	33 (29.2%)	39 (40.6%)	
None	80 (70.8%)	57 (59.4%)

**Table 3:** Univariate and multivariate analyses of risk factors with and OS in the training cohort.

Variables	HR (95% CI)	P value
**Univariate analyses**		
Age (years) (> 60 vs. ≤ 60)	1.470 (0.952, 2.270)	0.083
Gender (male vs. female)	0.851 (0.546, 1.326)	0.476
BMI	0.969 (0.928, 2.535)	0.146
Child–Pugh classification (B and C vs. A)	2.232 (0.927, 5.372)	0.073
Alcohol consumption (yes vs. no)	1.262 (0.812, 1.963)	0.302
Hepatitis B (yes vs. no)	0.284 (0.153, 0.525)	< 0.001
Hepatitis C (yes vs. no)	1.078 (0.553, 2.104)	0.825
Histologic grade (G3-4 vs. G1-2)	0.922 (0.590, 1.439)	0.720
Microvascular invasion (yes vs. no)	1.527 (0.917, 2.543)	0.103
AJCC tumor stage (III and IV vs. I and II)	3.317 (2.084, 5.278)	< 0.001
Risk score	2.714 (2.163, 3.405)	< 0.001
**Multivariate analyses**		
Hepatitis B (yes vs. no)	0.451 (0.227, 0.894)	0.023
AJCC tumor stage (III and IV vs. I and II)	1.917 (1.154, 3.182)	0.012
Risk score	2.163 (1.670, 2.800)	< 0.001

HR, hazard ratio; CI, confidence interval; OS, overall survival; BMI, body mass index; AJCC, American Joint Committee on Cancer.

**Table 4:** Univariate and multivariate analyses of risk factors with and OS in the validation cohort.

Variables	HR (95% CI)	P value
**Univariate analyses**		
Age (years) (> 60 vs. ≤ 60)	0.923 (0.507, 1.680)	0.794
Gender (male vs. female)	0.797 (0.436, 1.458)	0.461
BMI	1.023 (0.992, 1.055)	0.145
Child–Pugh classification (B and C vs. A)	0.967 (0.286, 3.274)	0.957
Alcohol consumption (yes vs. no)	0.558 (0.245, 1.267)	0.163
Hepatitis B (yes vs. no)	0.591 (0.270, 1.292)	0.188
Hepatitis C (yes vs. no)	1.051 (0.498, 2.221)	0.896
Histologic grade (G3-4 vs. G1-2)	1.778 (0.952, 3.323)	0.071
Microvascular invasion (yes vs. no)	1.156 (0.555, 2.407)	0.698
AJCC tumor stage (III and IV vs. I and II)	1.329 (0.691, 2.554)	0.393
Risk score	1.564 (1.200, 2.037)	0.001
**Multivariate analyses**		
AJCC tumor stage (III and IV vs. I and II)	0.569 (0.628, 2.331)	0.569
Risk score	1.756 (1.208, 2.554)	0.003

HR, hazard ratio; CI, confidence interval; OS, overall survival; BMI, body mass index; AJCC, American Joint Committee on Cancer.

### Nomogram based on the signature for predicting the OS of HCC patients

**Figure 5:**
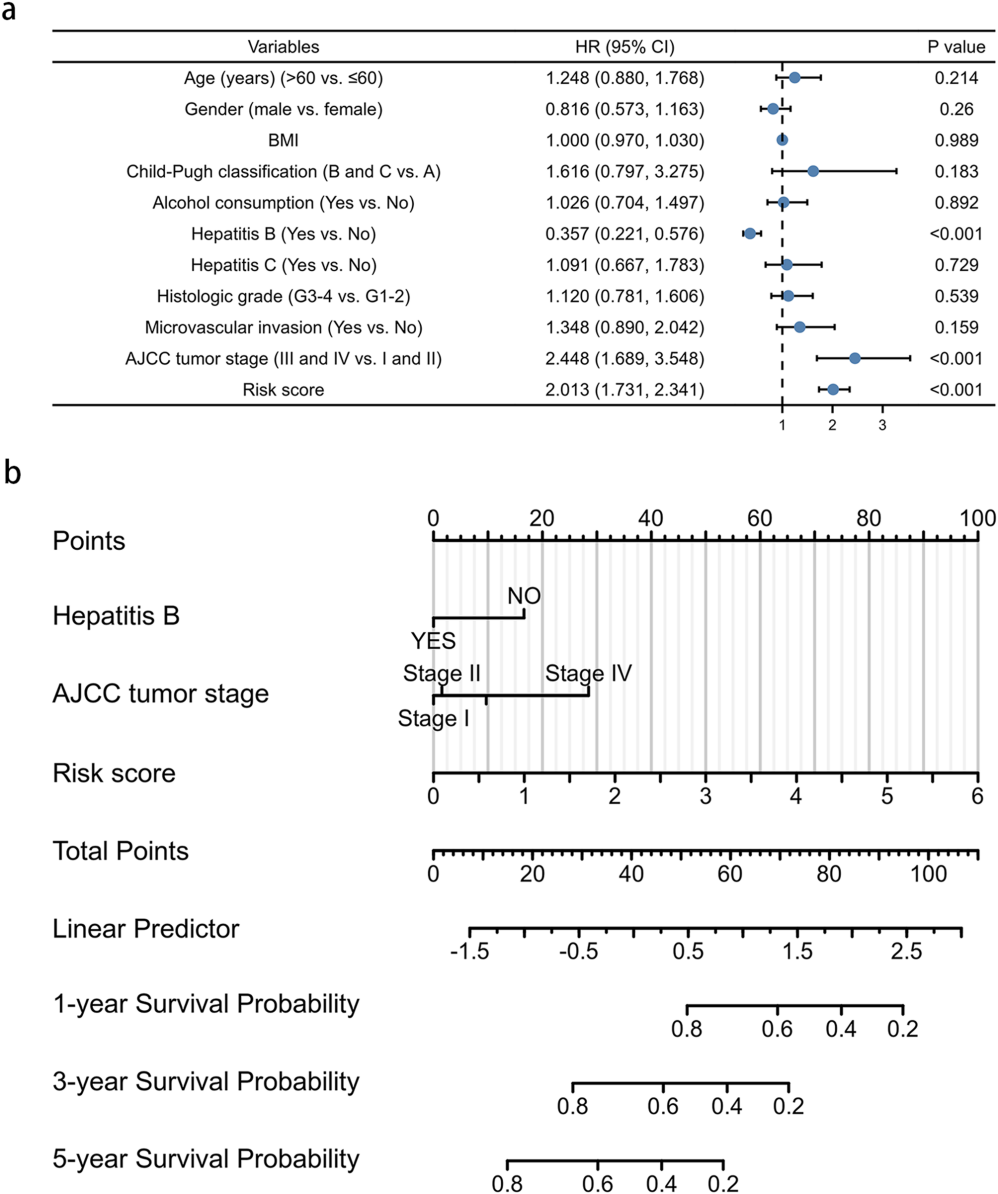
The prognostic values of pyroptosis-related lncRNA signature. (**a**) Multivariate Cox regression of patient characters and the signature in the whole cohort. (**b**) The nomogram constructed using patient characters and the signature.

In order to develop a more accurate model for prognosis prediction, univariate Cox regression analysis was performed in both cohorts (Fig. 5a). The pyroptosis-related lncRNA signature (hazard ratio 2.013, 95%; confidence interval 1.731–2.341), hepatitis B (hazard ratio 0.357, 95% confidence interval 0.221–0.576), and tumor stage (hazard ratio 2.448, 95% confidence interval 1.689–3.548) were risk factors for the prognosis of HCC patients. Proportional hazard assumption analysis was subsequently performed, and the results showed the variables did not violate the proportional hazard assumption (Supplementary Material 6). Then, a nomogram, including hepatitis B, tumor stage, and risk scores, was constructed to explore the probability of the lncRNA signature in predicting 1-, 3-, and 5-year survival in HCC patients with a C-index of 0.721. In this combined nomogram, the risk score model exerts the most excellent weight in predicting the 1-year, 3-year, and 5-year survival probability with a C-index of 0.711 (Fig. 5b). The findings identify that this novel pyroptosis-related lncRNA signature can act as a promising prognostic model for HCC patients.

### GSEA and functional enrichment analysis

**Figure 6:**
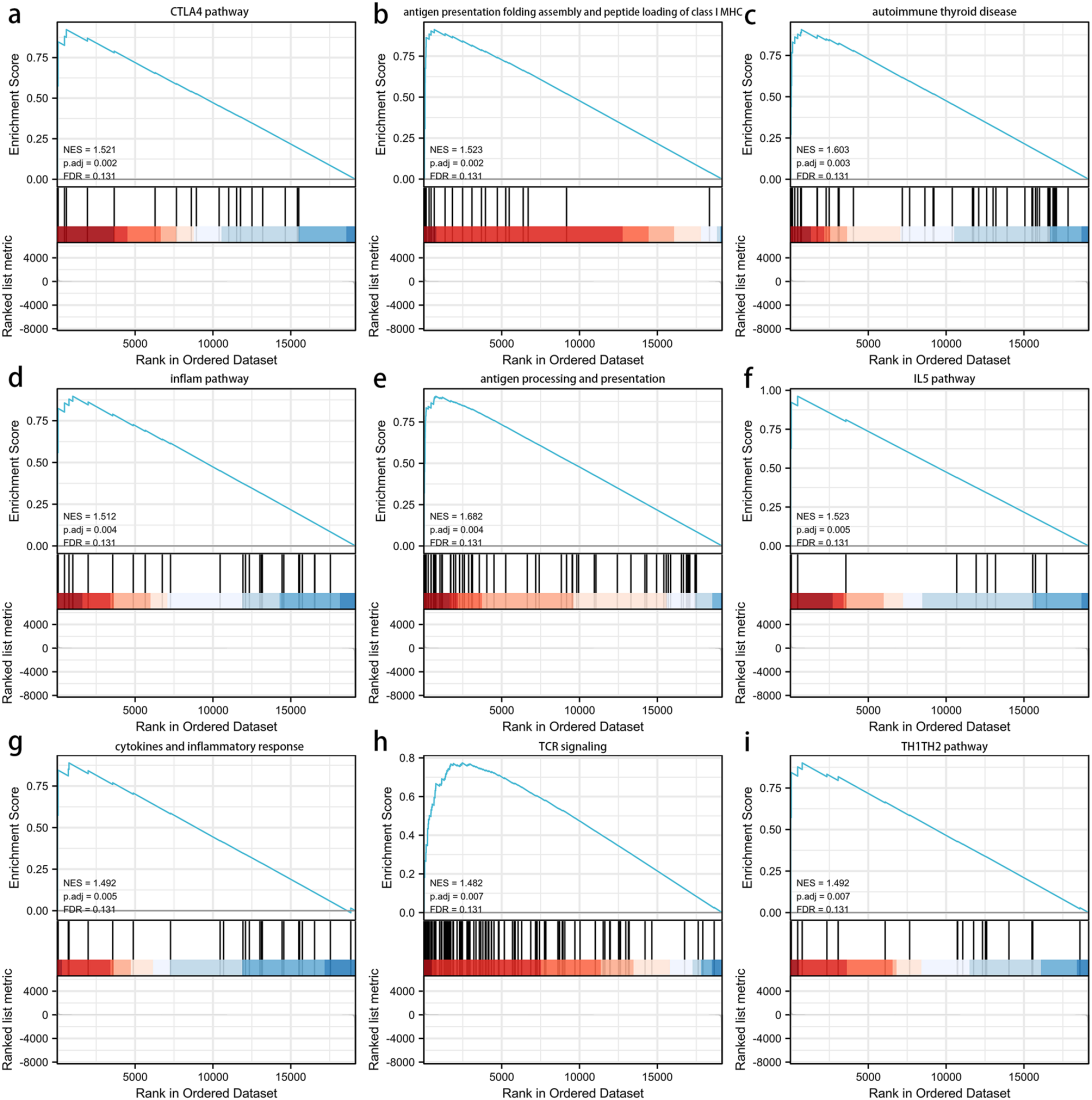
Gene set enrichment analysis (GSEA) about the pyroptosis-related lncRNA prognostic signature. (**a**) CTLA4 pathway. (**b**) antigen presentation folding assembly and peptide loading of class I MHC. (**c**) autoimmune thyroid disease. (**d**) inflam pathway. (**e**) antigen processing and presentation. (**f**) IL5 pathway. (**g**) cytokines and inflammatory response. (**h**) TCR signaling. (**i**) TH1TH2 pathway.

GSEA analysis was performed to explore the potential biological functions involved in the signature, using the differentially expressed genes between the low- and high-risk groups (Supplementary Material 7). Figure 6a–i shows the top 9 immune-associated signaling pathways, including the CTLA4 pathway, antigen presentation folding assembly and peptide loading of class I MHC, autoimmune thyroid disease, inflammatory pathway, antigen processing and presentation, IL5 pathway, cytokines and inflammatory response, TCR signaling, and TH1TH2 pathway. The results show that the pyroptosis-related lncRNA signature may be associated with multiple immune-related pathways.

### Associations between the signature and immune infiltration

**Figure 7:**
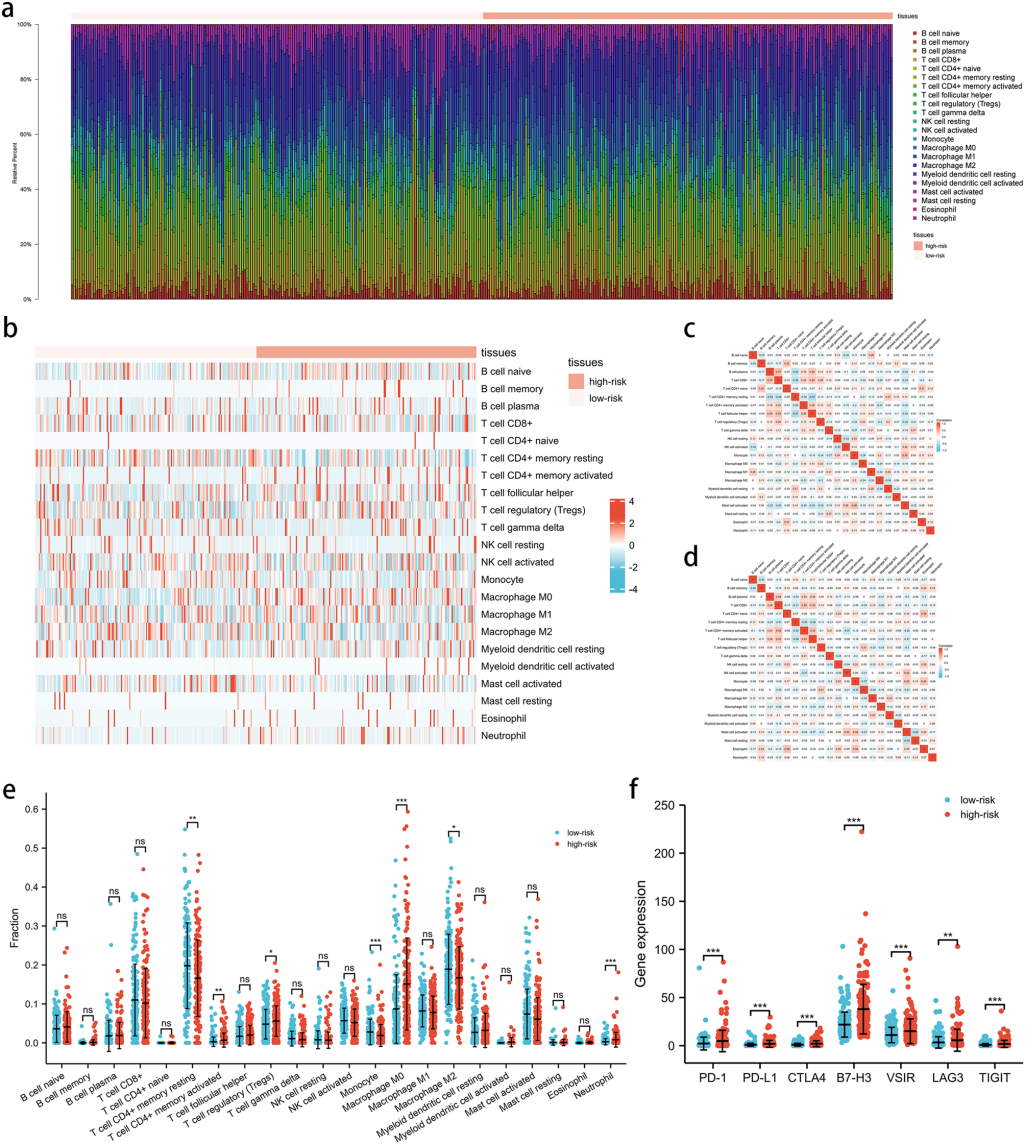
The interactions between pyroptosis-related lncRNA signature and immune regulation in HCC patients. (**a**) The barplot of the tumor-infiltrating cell proportions. (**b**) The heatmap of the tumor-infiltrating cell proportions. (**c**) Correlation matrix of immune cell proportions in the low-risk group. (**d**) Correlation matrix of immune cell proportions in the high-risk group. (**e**) Comparisons of immune cell proportions between the low-risk group and the high-risk group. (**f**) Comparisons of multiple immune checkpoints between the low-risk group and the high-risk group, including PD-1, PD-L1, CTLA4, B7-H3, VSIR, LAG3, TIGIT.

In order to explore the roles of the pyroptosis-related lncRNA signature in the immune microenvironment of HCC patients, the associations between the signature and immune infiltration cells were further investigated using the ESTIMATE algorithm (Supplementary Material 8). The proportions of various immune cells are shown in Fig. 7a, b, and the correlations between these immune cells in the low-risk and high-risk groups are shown in Fig. 7c, d. Among the immune cells, the CD4 + memory activated T cells, regulatory T cells (Tregs), M0 macrophages, and neutrophils were all increased in the high-risk group, while the CD4 + memory resting T cells, monocytes, and M2 macrophages were decreased in the high-risk group compared with the low-risk group (Fig. 7e). In addition, the associations between the signature and other immune checkpoints, including PD-1, PD-L1, CTLA4, B7-H3, VSIR, LAG3, and TIGIT, were also investigated. As shown in Fig. 7f, the immune checkpoints were significantly up-regulated in the high-risk group compared with the low-risk group. To conclude, the results indicate that the pyroptosis-related lncRNA signature may be associated with immune-related mechanisms and the response of HCC patients to immunotherapy.

## Discussion

In this study, prognostic pyroptosis-related lncRNAs in HCC were comprehensively investigated. A novel prognostic signature consisting of 9 pyroptosis-related lncRNAs (AC019080.5, AP003392.4, MKLN1-AS, AL031985.3, PCCA-DT, AC007128.1, LNCSRLR, AL445228.3, AC023157.2) was developed using LASSO Cox regression analysis and validated by the validation cohort. The subsequent functional analysis confirms the associations between the signature and multiple immune-related pathways. The results suggest that the identified signature has potential values in predicting the patient prognosis and managing immunotherapy for HCC patients.

Several studies have investigated the roles of lncRNAs in pyroptosis^13^. It is reported that lncRNA KCNQ1OT1 inhibits the miR-21 expression and subsequently promotes the caspase-1-dependent pyroptosis during cataract formation^14^. It is found that LncRNA MALAT1 increases the level of NLRP3 by up-regulating ELAVL1 expression, thus leading to cell pyroptosis in diabetic nephropathy^15^. It has been proven that LncRNA ANRIL up-regulates NLRP3 and IL-1β by increasing the expression of BRCC3, thus activating pyroptosis in uric acid nephropathy^16^. Furthermore, a recent study by Zhang et al. has demonstrated that lncRNANeat1 can promote the assembly of the NLRP3 and AIM2 inflammasome by directly binding to pro-caspase-1, thus accelerating caspase-1-dependent pyroptosis^17^. In terms of cancer, lncRNA RP1‑85F18.6 has been identified to promote pyroptosis by cleaving GSDMD in colorectal cancer^18^; LncRNA ADAMTS9-AS2 can activate NLRP3-mediated pyroptosis via sponging miR-223-3p in gastric cancer^19^. Another recent study has reported that SNHG7 inhibits NLRP3-dependent pyroptosis by targeting the miR-34a/SIRT1 axis in HCC^20^. At present, detailed knowledge about the roles of lncRNAs in regulating pyroptosis in HCC remains limited.

In the co-expression network, MKLN1-AS and AL031985.3 might be the most likely pyroptosis-related lncRNAs in the TCGA-HCC cohort. Studies have suggested that MKLN1-AS aggravates the progression of HCC by sponging miR-654-3p^21^. However, no studies have reported the potential role of AL031985.3 in HCC. Because of the great significance of MKLN1-AS, AL031985.3, and 7 other lncRNAs in HCC, future studies are encouraged to reveal the underlying mechanisms of these candidate lncRNAs in HCC biology, especially cell pyroptosis.

Our study shows significant relationships between the signature and many immune-associated signaling pathways. Patients with higher risk scores display a higher expression of multiple immune checkpoints, including PD-1, PD-L1, CTLA4, B7-H3, VSIR, LAG3, and TIGIT. Additionally, PD-1, a member of the B7/CD28 costimulatory receptor family, is expressed on multiple immune cells, including activated T cells, B cells, Tregs, and monocytes. Moreover, PD-L1 functions as the main ligand of PD-1^22^. Immunotherapy targetings PD-1 and PD-L1 have been developed, such as PD-1 inhibitors nivolumab and pembrolizumab targeting and PD-L1 inhibitors durvalumab and avelumab^23^. CTLA-4, a member of the immunoglobulin superfamily homologous to CD28, can inhibit T cells activity by competing with CD28^24^. Drugs targeting CTLA-4, such as ipilimumab and tremelimumab, have been developed and have displayed promising anti-tumor effects^25^. Here, this study suggests that patients with higher risk scores may benefit more from immunotherapy than those with lower risk scores.

However, this study has certain limitations. Firstly, cohorts in this study were mainly based on the TCGA database. Thus, real-world data are needed for further validations of the pyroptosis-related lncRNA signature in HCC patients. Secondly, this study failed to investigate the underlying immune-related mechanisms of the identified lncRNAs. Moreover, further studies should focus on the potential of the pyroptosis-related lncRNA signature as an indicator of immunotherapy.

## Conclusion

In summary, a novel pyroptosis-related lncRNA signature has been identified to precisely predict the prognosis of HCC patients. The signature is robustly associated with the tumor immunity, providing a personalized prediction model for the prognosis and immunotherapeutic response of HCC patients.

## Supplementary Information

Supplementary Information 1.

Supplementary Information 2.

Supplementary Information 3.

Supplementary Information 4.

Supplementary Information 5.

Supplementary Information 6.

Supplementary Information 7.

Supplementary Information 8.

## Data Availability

All data generated or analyzed during this study are included in this published article and supplement materials.

## Abbreviations

HCC: Hepatocellular carcinoma
lncRNAs: Long non-coding RNAs
TPM: Transcripts per kilobase million
LASSO: Least absolute shrinkage and selection operator
GSEA: Gene set enrichment analysis
TIMER: Tumor IMmune Estimation Resource
ANOVA: One-way analysis of variance
AUC: Area under curve
