# Supplementary figures and images for: Identification and validation of a novel pyroptosis-related lncRNAs signature associated with prognosis and immune regulation of hepatocellular carcinoma
Source: Sci Rep. 2022 May 25;12:8886. doi: 10.1038/s41598-022-13046-y (PMC9133103; doi:10.1038/s41598-022-13046-y)

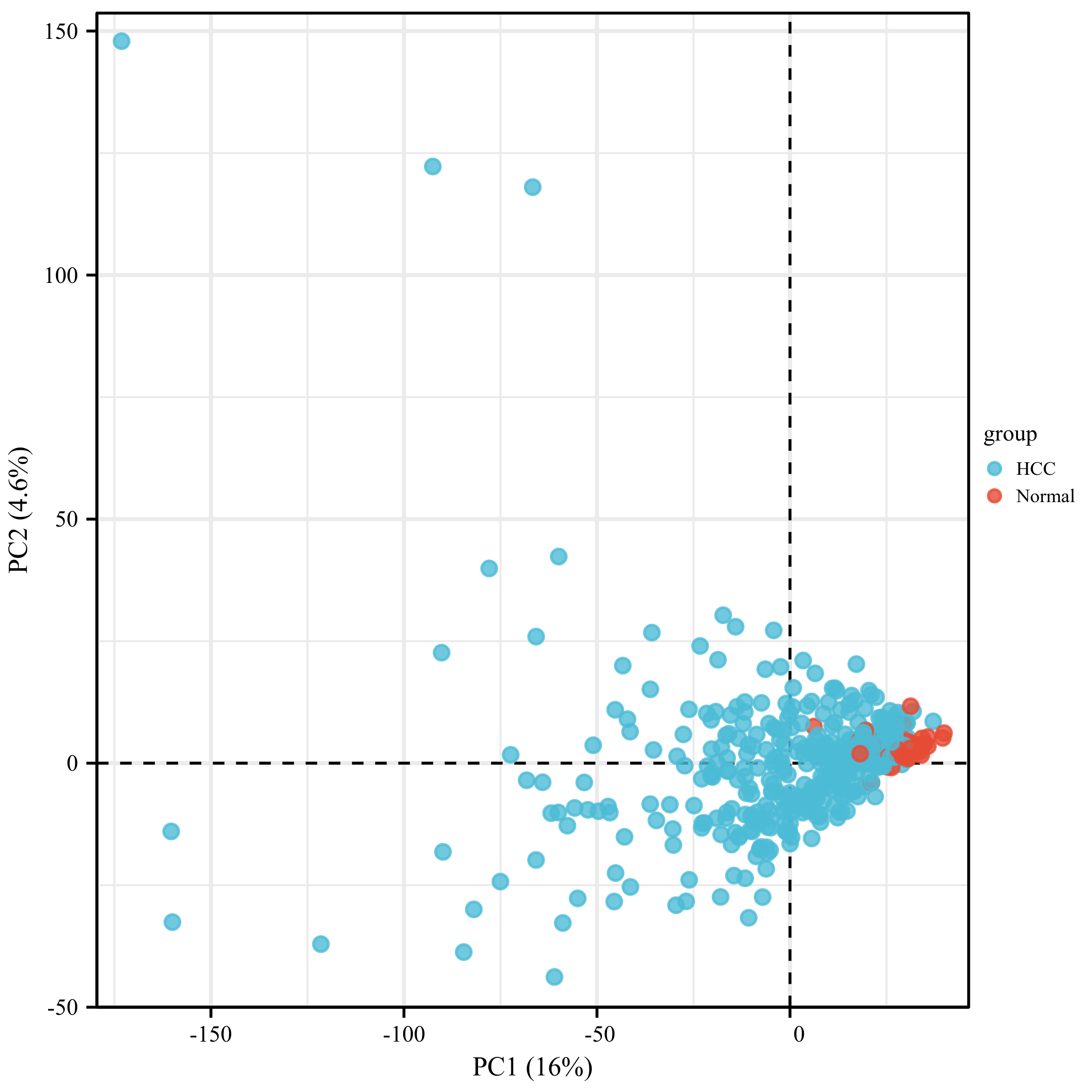


Supplment figure S1. The PCA analysis of 5372 selected lncRNAs

Supplement: Supplementary file 2 — Supplementary Information 2. [file 41598_2022_13046_MOESM2_ESM.docx]
