# Supplementary material for: Identification and validation of a novel pyroptosis-related lncRNAs signature associated with prognosis and immune regulation of hepatocellular carcinoma
Source: Sci Rep. 2022 May 25;12:8886. doi: 10.1038/s41598-022-13046-y (PMC9133103; doi:10.1038/s41598-022-13046-y)
Supplement: Supplementary file 5 — Supplementary Information 5. [file 41598_2022_13046_MOESM5_ESM.docx]

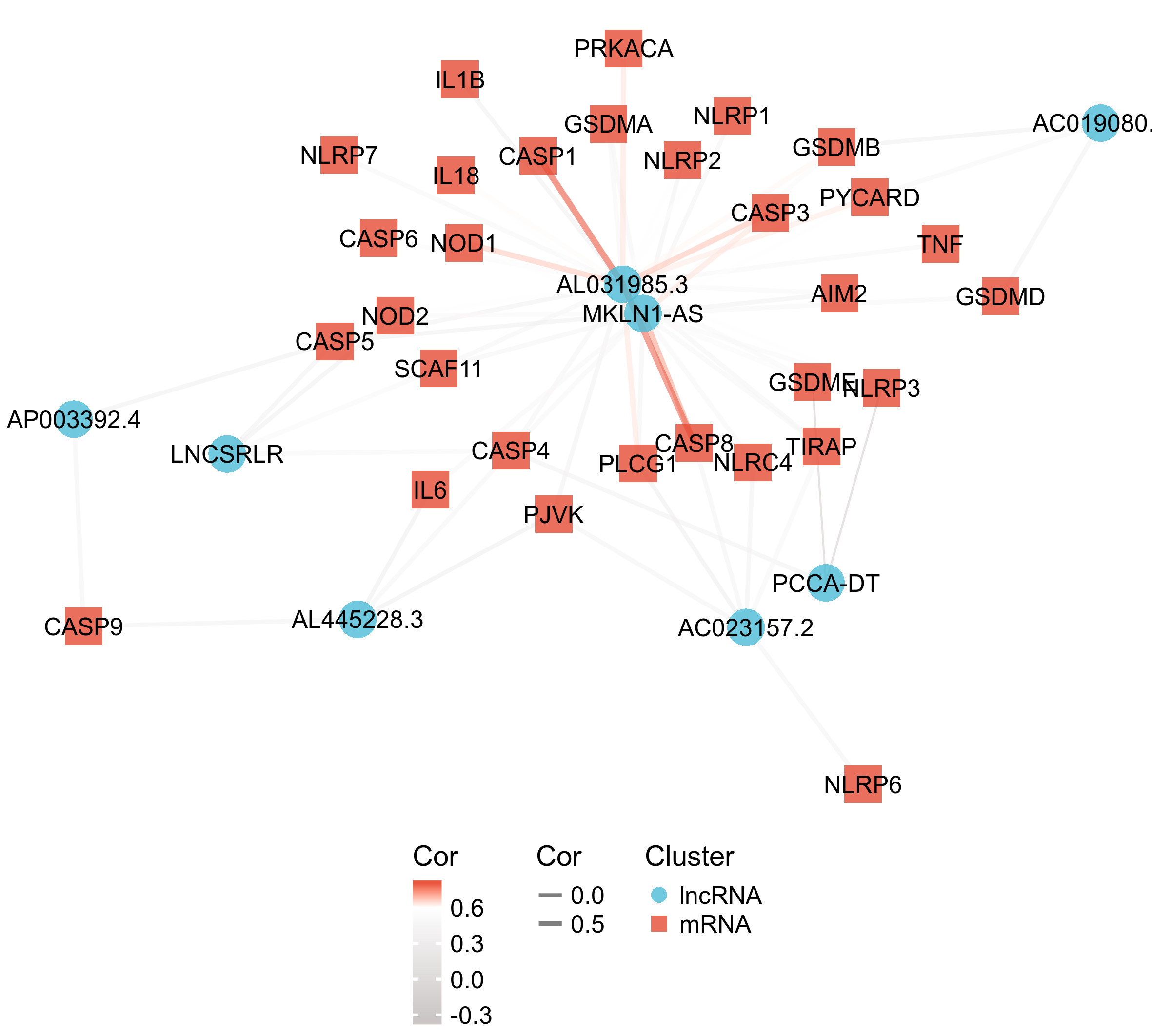


Supplement figure S2. mRNA-lncRNA co-expression network of the pyroptosis-related genes and the selected pyroptosis-related lncRNAs in the control group.
