# Supplementary material for: Identification and validation of a novel pyroptosis-related lncRNAs signature associated with prognosis and immune regulation of hepatocellular carcinoma
Source: Sci Rep. 2022 May 25;12:8886. doi: 10.1038/s41598-022-13046-y (PMC9133103; doi:10.1038/s41598-022-13046-y)
Supplement: Supplementary file 6 — Supplementary Information 6. [file 41598_2022_13046_MOESM6_ESM.docx]

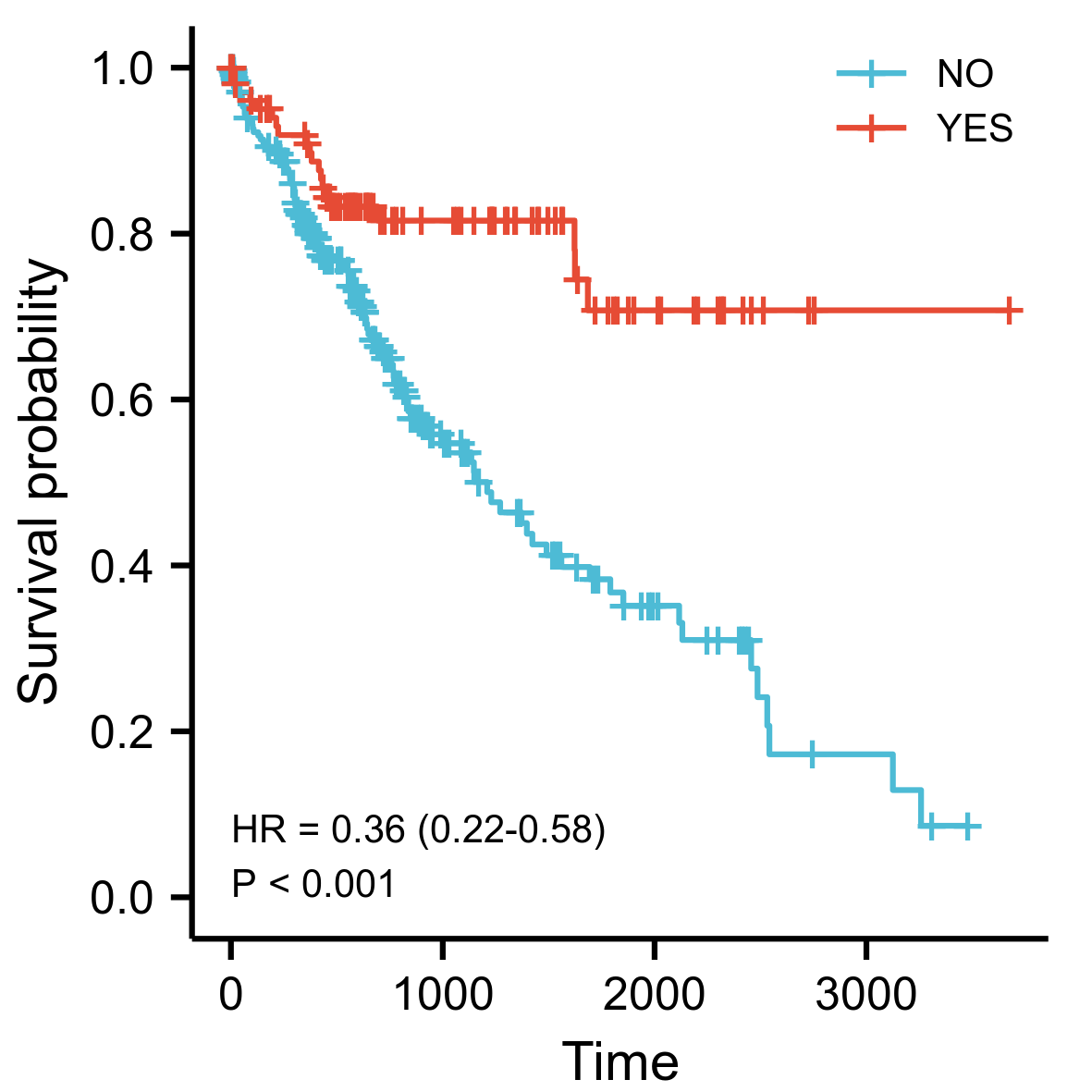


Proportional hazard assumption analysis of Hepatitis B

Supplement figure S3. Proportional hazard assumption analysis of hepatitis B

Proportional hazard assumption analysis of hepatitis B showed the variables did not violate the proportional hazard assumption by Kaplan-Miere method.


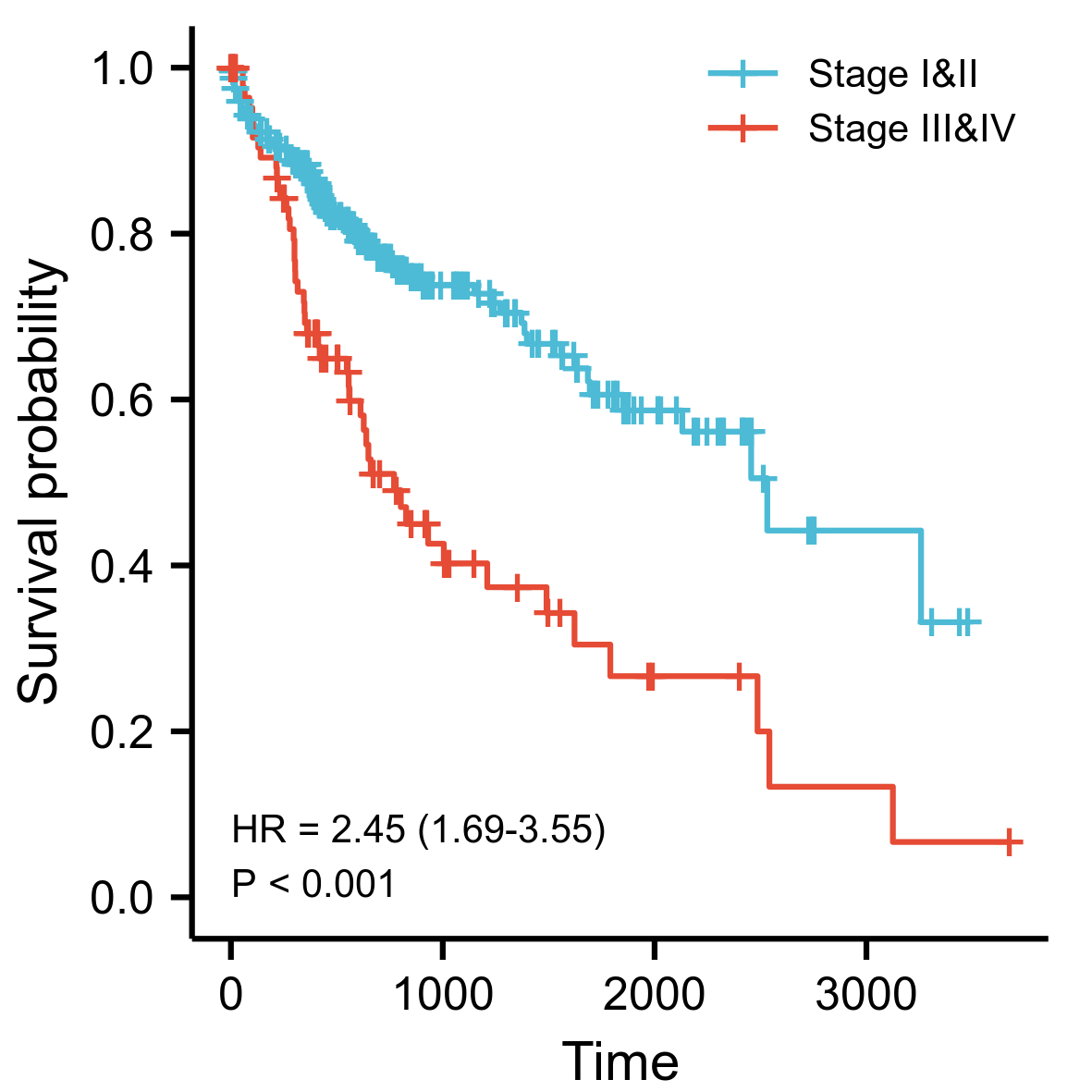


Proportional hazard assumption analysis of AJCC tumor stage

Supplement figure S4. Proportional hazard assumption analysis of AJCC tumor stage

Proportional hazard assumption analysis of AJCC tumor stage showed the variables did not violate the proportional hazard assumption by Kaplan-Miere method.
